# Security and Distribution, or Should You Care about Merely Possible Losses?

**DOI:** 10.1080/21550085.2018.1562532

**Published:** 2019-03-01

**Authors:** Kian Mintz-Woo

**Affiliations:** 1 Department of Philosophy, University of Graz, Graz, Austria; 2 University Center for Human Values and Woodrow Wilson School, Princeton University, Princeton, USA

**Keywords:** climate change, harms, loss and damage, risk, security

## Abstract

Jonathan Herington argues that harms can occur whether or not there is actually a loss. He claims that subjectively or objectively merely being at risk of losing access to basic goods is sufficient for lowering that individual’s well-being for the value of ‘security’. I challenge whether losing access to basic goods is sufficient to justify the introduction of this value. I also point to some issues in his interpretation of IPCC risk categories and the social science research he relies on.

Jonathan Herington (2017) argues that harms can occur whether or not there is actually a loss. In particular, he argues that merely being at risk either subjectively or objectively of losing access to basic goods is sufficient for lowering that individual’s well-being. This means that well-being, on this account, encompasses more than just the affective (as with hedonistic utilitarianism) or the desired (as with preference utilitarianism), but also another valuable relation between people and goods, which Herington calls ‘security’. Herington also claims that this has important policy implications – specifically, this provides extra justification for insurance against damages (of basic goods).

In this comment, I challenge whether losing access to basic goods is sufficient to justify the extra introduction of ‘security’ as an extra category of value, over and above the value of the basic goods themselves, as well as his claimed policy implications. I also point to some issues in his interpretation of IPCC risk categories and the social science research he relies on, comments which may be of broader interest.

Take ‘(mere) risking’ to be shorthand for situations where individuals are at a risk of losing access to (basic) goods but where that bad outcome does not eventuate. We have subjective and objective versions of (mere) risking, meaning that the risk is a function of an individual’s mental states or the possible future states of the actual world, respectively. I will argue that, for subjective risking, Herington’s account does not seem to add anything to simpler hedonistic or preference accounts and that, for objective risking, it is unconvincing that we ought to secure goods for possible counterparts over securing goods for actual people.

First, consider mere subjective risking. I actually think that Herington’s claims that this insecurity can be both unpleasant affectively and dispreferred seem right. However, these claims can be captured in standard hedonistic and preference accounts of well-being. So, in preference terms, assuming risk aversion and loss aversion, as is common in economics, we get that goods being insecure decreases utility or that, for most people, it is unpleasant to have goods threatened (both general goods and basic goods in particular). Hedonistic accounts of well-being would similarly find these unpleasant states negative in terms of well-being.

Herington thinks that there are extra costs not captured in these terms. His main piece of evidence is Mani, Mullainathan, Shafir, and Zhao’s (2013) excellent work, of which he writes that:

being at grave risk of impoverishment has a significant impact on cognition. For instance, in their study of poor farmers whose harvest is at risk of failing… they noted markedly diminished cognitive function relative to after their harvest has successfully been completed. (Herington, 2017, p. 189)

Again, I am sympathetic to Herington’s claim that risk of poverty can impact well-being (although I do not think this requires the introduction of ‘security’), but attributing the cognitive effect to stress or risk on the basis of this research requires misreading their piece; their claim is that *being poor* has an impact on cognition, not *being at risk of being poor*. They explicitly write that, while one might think that their effect was about anxiety over future earnings, in practice this ‘is not likely to be true in the case of sugarcane farming… sugarcane crop size can be readily estimated months before harvest’ (Mani et al., 2013, p. 979). Furthermore, 316 members of their sample were such that they were tested *after harvest* but before payment – these farmers would neither have objective nor subjective risk because they had already harvested their sugarcane and knew its value. The only variable was their *then*-poverty, and this subsample performed similarly to the full sample (p. 979). These points suggest that it was not any *risk* of poverty which generated the cognitive effects, it was *actual* poverty.

However, the more interesting and substantive claim that Herington makes is that fact-relative security, or not being (merely) *objectively* risked, might be valuable. Even if one is not actually harmed, if there is an increased probability of one’s goods being lost, then this is sufficient to be damaging for one’s well-being. He says it is possible that this can be explained in terms of preference satisfaction, since some might have preferences that our goods are not put at risk – even if they are not actually lost, but he admits that this is somewhat unsatisfying or unconvincing. I agree; it would be odd for people to have intrinsic preferences over potential states of affairs that they never learn about and which never eventuate.

Herington then takes a bolder approach, suggesting that we can appeal to distributional value in preventing insecurity. However, this distribution is not over members of the actual world since we are talking about *mere* risking which does not eventuate in actual negative outcomes; he writes that these are the cases where ‘the well-being of an individual is diminished by insecurity even if they are never aware of the risks and the relevant harms never materialize’ (190). Instead, the distribution is over the well-being of *possible* persons, or members of different possible worlds.^1^ Herington’s argument is that prioritarianism gives us reason to privilege the well-being of people who are less well-off, even merely possible people. This argument links to security because his conception of security is modal; a (basic) good is secure when one’s counterparts enjoy it in many accessible possible worlds, regardless of whether you come to enjoy it in the actual world.

The intuition seems to be that, if one’s future counterparts are able to enjoy a good, one (in the actual world) is thereby made more secure. Talk of transworld identity is highly vexing (Loux 1979); however, let us put the metaphysics aside and grant Herington that we can make sense of security for actual people in terms of the ability of counterparts to enjoy goods. I limit myself to two remarks.

First, if one granted that distribution matters for ethics over possible worlds, there is no reason that one would have to be prioritarian to make this claim. A (trans-world) utilitarian would be happy to distribute basic goods equally (or close to equally) over possible people since the utility that someone who has no or fewer basic goods gets from more basic goods will be considerably higher than the utility someone who already has them would get ceteris paribus. The prioritarian differs from the utilitarian when considering the distribution of goods in ‘pure’ terms like welfare or utility, not ‘impure’ terms like food, water, medical supplies, or consumption (for another way this pure/impure distinction matters for climate change, see Mintz-Woo, in press).

Second, I have a deeper evaluative worry. Although Herington wants to increase goods available to merely possible people, this means increasing the security of actual people and the security of actual people requires actual resources. This is exemplified by Herington’s own suggestion of increased insurance. Suppose that one were concerned about the risk to basic goods that may befall counterparts of ours in possible worlds; how would we compare these merely possible harms to actual harms? After all, there are far more possible people to worry about than actual people and if we spend resources making sure that possible people live as well as possible (or at least live a sufficiently good (possible) life), that would require huge costs to actual people. Even if we wanted to secure goods for some relatively small number of possible people, that would entail costs for actual people. After all, insurance that does not pay out costs money for consumers, and if insurance is not purchased, this money could be used to finance consumption in the real world. Would we really rather they were made secure in the sense that their *merely* possible counterparts lived satisfactorily as opposed to their *actually* having better lives in the real world? I find this rather implausible.

One more tangential point about the paper which may be helpful for many environmental philosophers. While Herington is absolutely right that there is significant difficulty in determining the probabilities of various harms due to climate change, especially when downscaled regionally or locally, and he is also right that this does not preclude doing our best to assess probabilities given our epistemic situation (Roser, 2017), he seems to mischaracterize something about IPCC language regarding risk. Casual readers of the Summaries for Policymakers (SPM) might also be subject to the same mistake. Herington reads the SPM for the Fifth Assessment Report Working Group 2 (IPCC, 2014, pp. 19–20)^2^ and writes the following:

When considering non-economic harms, the IPCC is even less confident, expressing impacts in extremely qualitative terms. For instance, in considering the human health impacts of climate change, the IPCC tentatively suggests that a business as usual model will lead to a ‘greater likelihood of injury, disease, and death due to more intense heat waves and fires’ and that ‘the magnitude and severity of negative (health) impacts are projected to increasingly outweigh positive (health) impacts’. (Herington, 2017, p. 186)

Herington takes these perhaps circumspect-sounding terms to indicate lack of confidence, but this is not the case, as can be seen by considering the context of these quotations and consulting the risk guidance note from Mastrandrea et al. (2010). The SPM context for these quotes reveals that the claim about injury, disease, and death is given ‘very high confidence’ and the claim about the comparison of health impacts is given ‘high confidence’ (IPCC, 2014, pp. 19–20). Consulting the guidance note, we can see that this apparently qualitative language is given systematic and, indeed, confident definitions (very high confidence indicates ‘high agreement, robust evidence’ and high confidence indicates ‘high agreement, medium evidence’ or ‘medium agreement, robust evidence’) (Mastrandrea et al., 2010, Figure 1). Although this use of risk language is not transparent for a casual reader of the SPMs or IPCC reports more generally, I hope that these points are helpful to readers of this journal.

## Notes

1. I suspect that Herington has in mind branching future possible worlds with objective chance built into reality in such a way that the present determines which worlds are accessible and that what he really means is in *future possible worlds* which are sufficiently *close to the actual world* in the present. While objective chance is controversial (and, no, quantum indeterminacy will not be sufficient to guarantee macro indeterminacy), we can grant him these metaphysical assumptions here since discussion of them would take us too far afield.

2. Unfortunately, there is a typographical error and, instead of ‘pp. 19–20’, ‘p. 1920‘ is written (Herington, 2017, p. 186).
